# A Helical tomotherapy as a robust low‐dose treatment alternative for total skin irradiation

**DOI:** 10.1002/acm2.12579

**Published:** 2019-04-29

**Authors:** André Haraldsson, Jens Engleson, Sven Å. J. Bäck, Silke Engelholm, Per E. Engström

**Affiliations:** ^1^ Department of Hematology, Oncology and Radiation Physics Skåne University Hospital Lund Sweden; ^2^ Medical Radiation Physics Department of clinical sciences Lund University Lund Sweden

**Keywords:** fungoides, helical, mycosis, skin, tomotherapy, total skin irradiation

## Abstract

Mycosis fungoides is a disease with manifestation of the skin that has traditionally been treated with electron therapy. In this paper, we present a method of treating the entire skin with megavoltage photons using helical tomotherapy (HT), verified through a phantom study and clinical dosimetric data from our first two treated patients. A whole body phantom was fitted with a wetsuit as bolus, and scanned with computer tomography. We accounted for variations in daily setup using virtual bolus in the treatment plan optimization. Positioning robustness was tested by moving the phantom, and recalculating the dose at different positions. Patient treatments were verified with *in vivo* film dosimetry and dose reconstruction from daily imaging. Reconstruction of the actual delivered dose to the patients showed similar target dose as the robustness test of the phantom shifted 10 mm in all directions, indicating an appropriate approximation of the anticipated setup variation. *In vivo* film measurements agreed well with the calculated dose confirming the choice of both virtual and physical bolus parameters. Despite the complexity of the treatment, HT was shown to be a robust and feasible technique for total skin irradiation. We believe that this technique can provide a viable option for Tomotherapy centers without electron beam capability.

## 1 INTRODUCTION

Mycosis fungoides is a rare form of non‐Hodgkin T‐cell lymphoma mainly affecting the cutaneous tissue. The incidence is around three per 1000 000 person‐years in Sweden. Early clinical manifestation is characterized by limited plaques, and later by tumors, widespread ulceration, and systemic involvement which can cause severe itching. A number of treatments are available, but none induces long‐term remission, and treatment is therefore often regarded as palliative despite long survival. Mycosis fungoides has been treated with radiotherapy since the 1960s,^1^ and Total Skin Electron Beam Therapy (TSEBT) is considered the standard treatment today.^2^, ^3^, ^4^ Traditionally, a prescribed dose of 30–36 Gy over 6–10 weeks has been recommended,^4^ but recently, doses as low as 10–12 Gy have been used for step‐wise short‐term palliation.^5^ Lower‐dose regimes have two main advantages: the treatment time is shorter, and the toxicity lower, which allows re‐irradiation. To cover as large an area of the skin as possible, TSEBT is administered with the patient standing on a rotating platform or at several fixed positions at an extended source to skin distance (SSD) of 3–8 m using a beam degrader. TSEBT offers good short‐term remission and few reported cases of severe toxicity.^4^ However, it is not possible to irradiate all the cutaneous tissue with this technique, and several patch fields are needed, raising questions regarding over‐ and underdosage at the field junctions. In addition, lead shielding of genitals, eyes and lips is necessary, making the technique cumbersome.

An alternative mode of treatment is total skin irradiation (TSI) with helical tomotherapy (HT),^6^ a technique combining couch translation and continuous gantry rotation. With this technique,^7^, ^8^ targets as long as 135 cm can be irradiated in one field.^9^ Treatment of longer targets requires the field to be split but still allowing the whole skin to be treated on one occasion. Furthermore, skin folds can be covered by defining them as target in the optimization, and organs such as the eyes, genitals and lips can be avoided. For TSI with HT, the patient can lie down in supine position during the entire treatment as opposed to standing. This technique can be of value for centers without capability of electron treatment of the entire skin, but also for partial irradiation of the skin. A few studies have previously reported on TSI with HT,^7^, ^10^, ^11^ In this work, we evaluate the robustness of TSI with HT and implementation of virtual and physical bolus in the form of a wet suit and verify phantom data with clinical data.

The feasibility, deliverability, and assessment of robustness for the first two patients treated at our clinic is described.

## 2 MATERIALS AND METHODS

### 2.A Overview

Several issues regarding patient positioning, treatment planning, and delivery needed to be addressed before commencing clinical TSI. In order to achieve a geometrically robust treatment plan, a virtual bolus was designed and applied in the optimization. To test the robustness of the treatment plan, a whole body phantom was shifted and recalculated in the planning system for several positions and verified with dose measurements. Since the dose delivery of TSI is extremely complex, given that only tangential beams are used, the dose calculation accuracy of the treatment planning system (TPS) was verified for both surface dose and scattered central dose. During treatment, the patients were fitted with a wet suit of Neoprene, which is a non‐tissue equivalent material of unknown electron density and hence the bolus effect of Neoprene needed to be carefully evaluated. *In vivo* measurements were performed to verify the dose to the skin, on both patients and phantom.

### 2.B Patient characteristics

The first patient was a 72‐yr‐old male diagnosed with MF 2003. He had previously received radiotherapy with kilovoltage x‐ray on several occasions and had also been treated with PUVA + Methotrexate, Neotigason, and Targretin. At the time of TSI he had patches and plaques covering more than 10% of the body surface.

Patient 2 was a 43‐yr‐old female diagnosed with MF in 2007. She had been treated with TSEBT in Cairo in 2008, 32 Gy in 24F and she had also been given 35 treatments on different lesions with kV x‐ray. She had received systemic therapies with Interpheron, Tagretin, Neotigasone, and Methotrexate. At the time of treatment, she had patches and plaques covering more than 10% of the body surface. The TSI treatment was followed by a haploidentical allogenic bone marrow transplant with her 18‐yr‐old daughter as donor 3 weeks after the last fraction.

### 2.C Phantoms and detectors

A number of phantoms and detectors were used in this study.

- An anthropomorphic whole body phantom, PH‐2B CT (PBU‐60) (Kyoto Kagaku, Kyoto, Japan), with and without a neoprene suit. The density of simulated soft tissue of the phantom is 1.061 g/cm^3^, with a relative electron density of 0.975. The weight is 50 kg and the length 165 cm. The phantom includes relevant organs such as a lung cavity and a synthetic skeleton.
- A TomoTherapy phantom (Accuray Inc., Madison, WI, USA), which is a cylindrical Solid Water (RMI Gammex) phantom with varying density plugs, inserts for an A1SL ion chamber, and a removable midsection for film dosimetry.
- Solid Water slabs, size of 550 × 150 mm with thicknesses of 5–50 mm.
- A Delta4 1042 cross‐plane PMMA diode array detector with a density of 1.19 g/cm^3^ and relative electron density of 1.16 (Scandidos, Uppsala, Sweden).
- Two separate Exradin A1SL ion chamber (Standard Imaging Inc., Middleton, WI, USA).
- Gafchromic EBT3 film (ISP, Wayne, NJ, USA) together with evaluation software FilmQA Pro (Ashland, Bridgewater, NJ, USA) and an Epson 4990 flatbed scanner (Seiko Epson Corporation, Nagano, Japan).

### 2.D Immobilization and computed tomography (CT)

#### 2.D.1 Phantom

**Figure 1:**
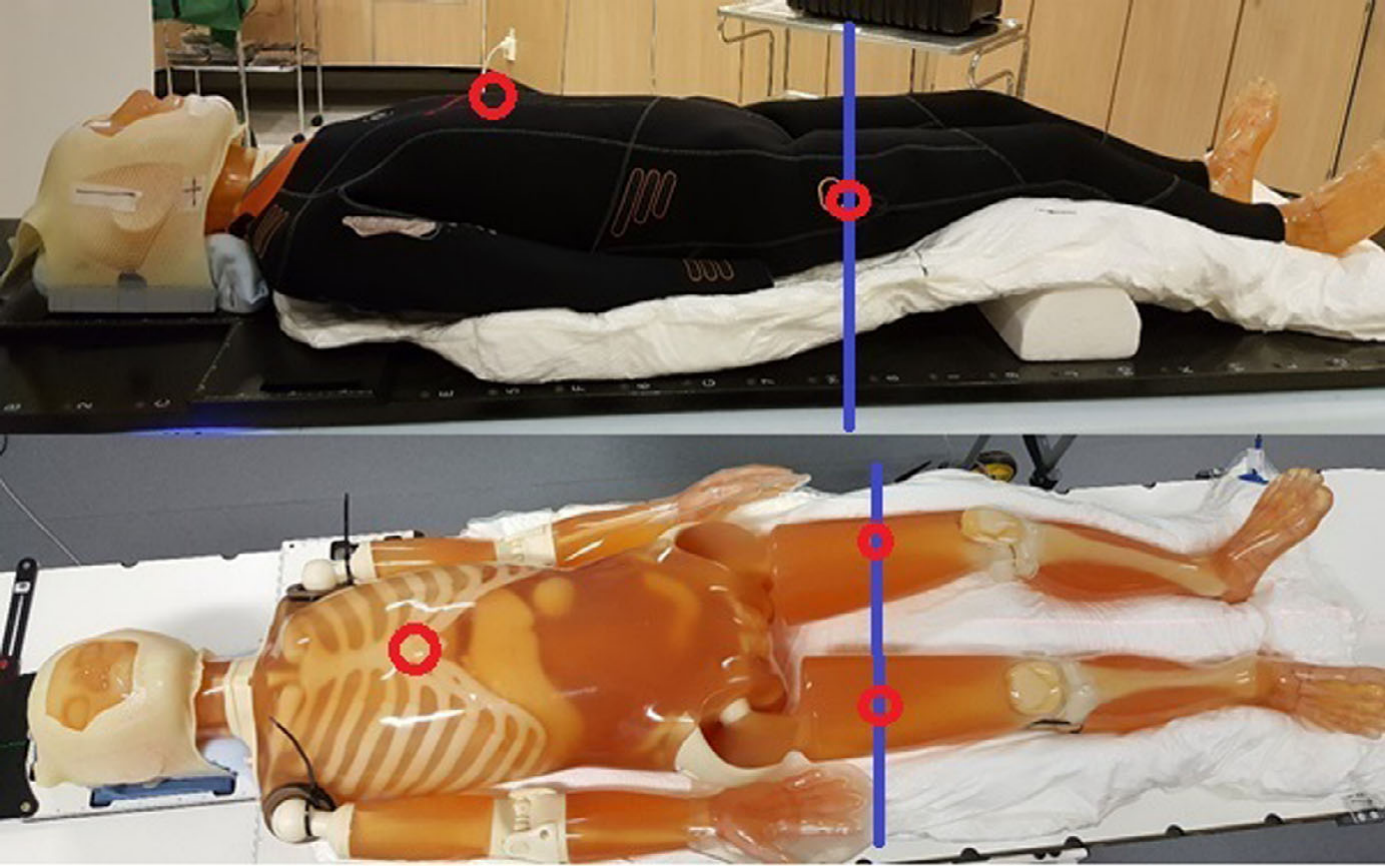
The anthropomorphic whole body PBU‐60 phantom, immobilized by a large vacuum cushion, with and without the wetsuit, showing the thermoplastic mask and support under the knees. Red circles mark the position of the internal reference points for the two plans and the blue line marks the position of the field junction.

Prior to CT, the PBU‐60 phantom was immobilized with a large vacuum cushion (VacFix, Par Scientific A/S, Odense, Denmark), an individually molded neck rest, and a 3‐point open‐face thermoplastic mask (Orfit Industries, Wijnegem, Belgium). Arms and hands were placed close to the trunk, the knees were slightly flexed, and the feet immobilized by the vacuum cushion, as shown in Fig. 1. Fiducial markers and tape were placed on the phantom marking the position of the lasers and the field junction position on the thighs. Since the phantom was longer than 135 cm, it was scanned in two parts, using a Siemens CT Somatom Definition Plus Scanner (Erlanger, Germany), with the wet suit in place. The first scan covered vertex to the thigh in head‐first supine (HFS) position, and the second from the toes to the upper thigh in feet‐first supine (FFS) position. Between scans, the vacuum cushion was rotated 180° and the head and neck immobilization removed. The two scans were performed with a slice thickness of 5 mm and overlapped by approximately 15 cm.

#### 2.D.2 Patients

The patients were immobilized and scanned following the same procedure as the phantom, but with a 5 point open‐face thermoplastic mask (Orfit Industries, Wijnegem, Belgium) and added wet suit socks, hood, and gloves. Patients were CT‐scanned wearing the full wet suit in order to assess and account for anatomical effects from the tight fitting suit both in treatment planning and in image registration during treatment. The body mass index (BMI) of patient #1 was 24, and 28 for patient #2.

### 2.E Planning and optimization

#### 2.E.1 Phantom

The results of both scans were exported to the TPS Eclipse (Varian Medical Systems, Palo Alto, CA, USA) and oncology information system Aria (Varian Medical Systems, Palo Alto, CA, USA) where the target and all relevant organs at risk (OARs) were delineated. The clinical target volume (CTV) was defined as the entire area of the skin to a depth of 5 mm, excluding the genitals, lips, and eyes. The planning target volume (PTV) was defined as a 5 mm isotropic expansion of the CTV.

The prescribed dose was defined as 12 Gy in 6 fractions for the phantom. Optimization was performed in the TomoTherapy treatment planning software (Accuray Inc., Madison, WI, USA) using fine resolution (1.95 × 1.95 mm^2^) for both optimization of the treatment plan and for final dose calculation. Planning parameters were set to a pitch of 0.200,^12^ a field width of 5 cm, a modulation factor of 2.3, and a minimum of 500 iterations. To aid in optimization, several internal blocking structures were defined. These structures were cropped from the PTV inwards by 5, 15, and 30 mm, where the 30‐mm structure were set to completely block the fluence. This procedure prevented all except tangential beams from entering the patient/phantom, thus reducing the dose to deep‐lying organs. The aim of planning optimization was to achieve the prescribed dose to cover 60% of the PTV, and a minimum of 95% of the prescribed dose would cover 95% of the PTV. The shape of the blocking structures was modified until target coverage was deemed acceptable.

The field junction was designed to be robust for uncertainties in patient positioning. A dose gradient was achieved on both CT sets by contouring a junction structure centered at the junction markers in the longitudinal direction. We started with a 4 cm long junction structure and then adjusted the length until coverage was acceptable. The junction structure was set as a target structure, without setting the structure *in use* and with an overlap priority higher than any other target structure. This achieves a similar effect as cropping the PTV. In combination with optimization with fixed jaws, this procedure creates a dose fall‐off at the field junction. The dose distribution from both treatment plans were imported to Eclipse for dose summation. In addition, we used the delivery quality analysis (DQA) module to reposition and recalculate the upper body of the PBU‐60 phantom by 5 mm and by 10 mm in all directions, where the resulting dose matrices where exported to Eclipse and added together with the lower body. The repositioned dose distribution was evaluated to assess the robustness of the junction under positioning deviations.

#### 2.E.2 Patient

The prescribed dose was defined as 12 Gy in six fractions for the first patient whereas the second patient was prescribed 20 Gy in ten fractions. Planning and optimization were performed using similar planning parameters as in the phantom study, with several internal blocking structures to prevent dose to internal organs such as bone marrow.

### 2.F Virtual bolus

**Figure 2:**
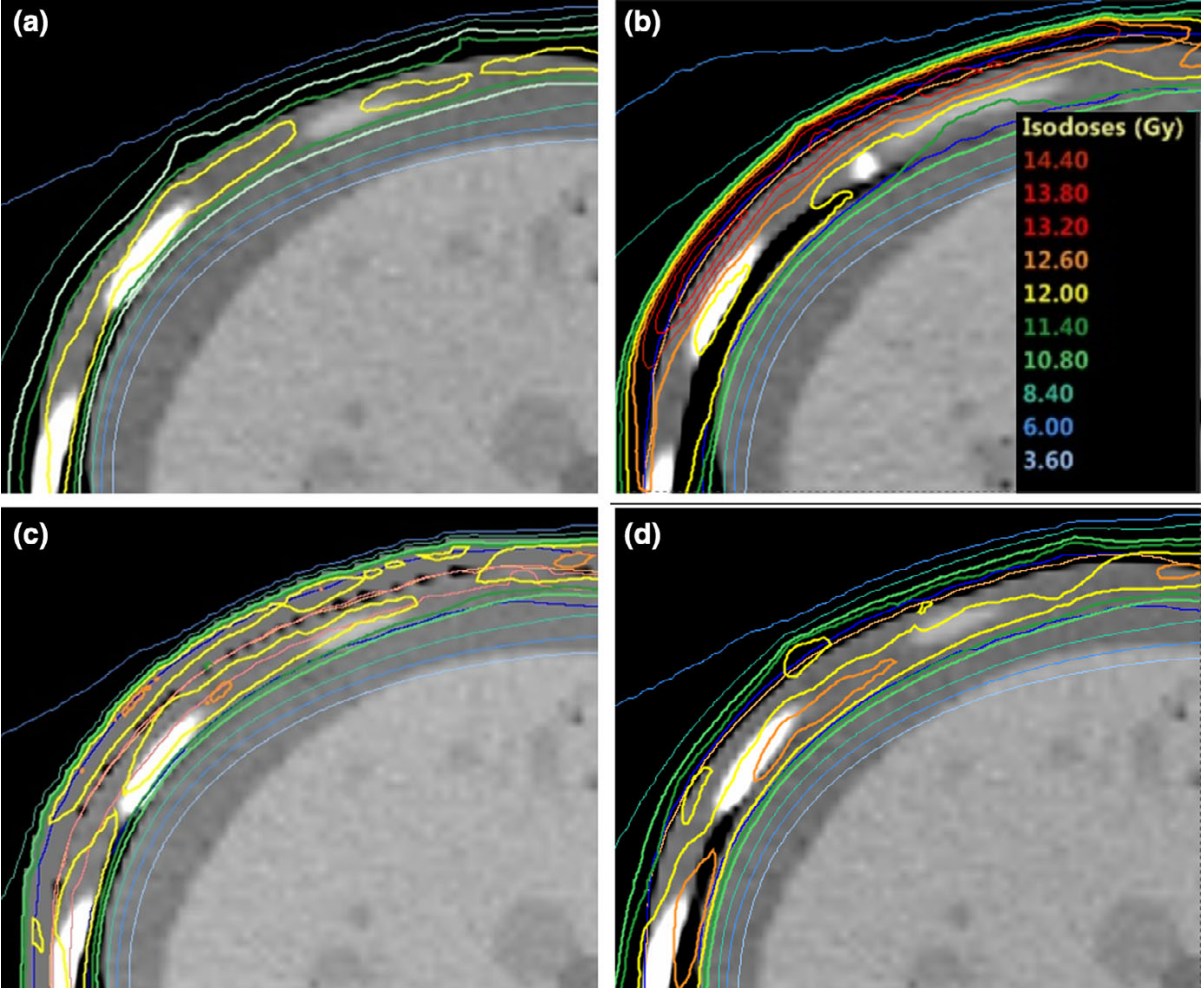
Difference in skin dose to patient 1 when the position is shifted by 5 mm lateral from the planned position for virtual bolus with a density of 0 g/cm^3^ (b) and a virtual bolus with a density of 1 g/cm^3^ (d) as compared to original position (a, c) when the treatment is planned without a virtual or physical bolus.

A virtual bolus was used to prevent over‐optimization of the fluence in air, due to expansion of the PTV outside the body. The wet suit was replaced by virtual bolus in the optimization since the fit of the suit varied from day to day. Targets very close to the tissue–air border causes the TPS to compensate the fluence to achieve full dose in the build‐up region and in the air surrounding the body. If the patient is not perfectly aligned during treatment, the patient may receive a dose well above that prescribed during treatment (Fig. 2). This can be managed by using a virtual bolus.

#### 2.F.1 Phantom

With the whole body phantom, optimization tests were performed in the TPS using a varying bolus density of 0, 0.4, and 1.0 g/cm^3^. The thickness of the virtual bolus was 8 mm, that is, the PTV with an additional 3 mm margin, as suggested by Moliner.^13^

#### 2.F.2 Patients

Although the patients were CT‐scanned wearing the full wet suit, a virtual bolus of water of specified density was still added in the TPS for two reasons; to account for daily variations caused by the fit of the wet suit and secondly, to replace the unconventional bolus material of neoprene with a material of well‐known dosimetric properties. The bolus was applied uniformly over the entire skin.

### 2.G Physical bolus

#### 2.G.1 Phantom

A 7 mm thick foamed neoprene (polychloroprene), wetsuit (AquaLung Dive, US) was used as a physical bolus for the PBU‐60 phantom. A wetsuit was chosen as bolus since it can be made to cover almost the entire body, has a uniform thickness and no metal components. The wetsuit covered the entire phantom except hands, feet and head.

#### 2.G.2 Patients

For the patients, a hood, gloves, and socks of neoprene were also added. In addition, patient #2 had a 5 mm water equivalent bolus (Superflab bolus, Radiation Products Design Inc., Albertville, MN, USA) covering eye lids and forehead, due to lesions in the face. The hood was open in the face but covering chin, ears, and above hair line.

### 2.H Robustness tests

#### 2.H.1 Phantom

To verify the geometric robustness of the technique using a virtual bolus of 0.4 g/cm^3^ of 8 mm thickness together with a 7 mm neoprene bolus, the final treatment plans were exported to the built‐in module for DQA. This module can be used to recalculate treatment plans for different geometries and phantoms. In this study, the treatment plan was recalculated for the upper body omitting the virtual bolus. The PBU‐60 phantom was then repositioned by ±10 mm in the longitudinal, vertical, and lateral directions. The resulting dose matrices were exported to the Eclipse TPS for summation and comparison.

#### 2.H.2 Patients

To assess the robustness of the patients' treatment, data acquired from the daily megavoltage computed tomographic (MVCT) was used to recalculate the delivered dose and compare it to the original treatment plan. For the first six fractions, we recontoured the CTV on the daily MVCT images and recalculated the treatment plans. The calculations were performed in the TomoTherapy Planned Adaptive module, and the registration data from the treatment was used to match the images. The obtained dose volume histograms were compared to the original plan and to the robustness calculations of the whole body phantom as described in the previous section. In addition to calculations, we measured the skin dose *in vivo* with film at the first fraction. Because the position of the junction of the first patient at the hip differed from the PBU‐60 phantom, the dose across the field junction was verified by shifting translating the upper body 5 mm in six directions (±x, ±y, ±z) and the resulting dose matrices was summed in Eclipse for verification.

### 2.I Film measurements

#### 2.I.1 Skin dose

##### Phantom

To verify the delivered surface dose, 24 Gafchromic EBT3 film strips of 2 × 3 cm were placed on the surface of the phantom beneath the wet suit, and distributed over the entire body. The PBU‐60 phantom was positioned and irradiated first in the HFS position and then in the FFS position, with field edge matching at the mid‐thigh position. One strip from each sheet was irradiated with 2 Gy at a depth of 1.5 cm in Solid Water and used as a dose reference.

##### PATIENTS

To assess the patient dose to the skin at treatment, we performed *in vivo* dosimetry with EBT3 film at the first fraction. At least 20 film strips of 1 × 1.5 cm^2^ were taped on several positions on the patients' skin. A reference irradiation was performed at 2 Gy in solid water at 1.5 cm depth with a minimum of 20 cm backscatter.

#### 2.I.2 Bolus measurement

The bolus effect of the neoprene wet suit fitted on the PBU phantom was quantified by paired film measurements, where film where placed beneath the wetsuit for the first measurements and replaced for the second measurement without wetsuit. In addition, a strip of film was placed on a 20 cm thick Solid Water slab and irradiated with and without a 200 × 200 × 7 mm^3^ square of neoprene to measure the buildup effect of neoprene. We compared the two measured groups using Wilcoxon signed‐rank test.

#### 2.I.3 Film evaluation

Prior to each film measurement, a strip of film from the same sheet as that used for measuring was irradiated with 2 Gy at depth of 1.5 cm in Solid Water with at least 20 cm backscatter and the TomoTherapy set in verification mode, that is fixed gantry with no couch travel. The films were scanned with an Epson 4990 flatbed scanner at least 24 h after exposure, and evaluated using the FilmQA Pro software using the reference film strip for dose normalization. The films strips were covered with a glass sheet, and scanned with a 16‐bit pixel value, and 5 × 5 mm region of interests (ROIs) for averaging. The same evaluation procedure was used for both phantom and patient measurements. Film and dose calibration were verified using film strips at depths of 1.5, 5, and 10 cm in solid water slabs, irradiated twice at different occasions. The values obtained were compared to the dose measured with an A1SL ion chamber at corresponding points of measurements. Surface dose measurement were compared to the calculated dose in the TPS, obtained with the plan recalculated without the virtual bolus.

### 2.J Ion chamber measurements

The optimized plan, restricted to only tangential irradiation was delivered to the cylindrical Tomotherapy phantom, to verify the accuracy of the dose calculation algorithm of the TomoTherapy TPS, at depths far from the main interaction sites. The plan was optimized with the phantom surface as target, to 4 Gy per fraction, and with margins and a virtual bolus specification identical to those used for the whole body phantom. The depth dose was measured using two A1SL ion chambers at several positions in the phantom and compared to the dose calculated by the TPS.

### 2.K Diode array measurements

Dose verification was also performed using the Delta4 diode array detector placed at several locations to cover the entire irradiation volume of the treatment plan. The measured dose was compared to the planned dose using gamma evaluation.^14^ Quality control (QC) acceptance criterion was set to 90% pass rate using 2 mm distance to agreement, 3% dose difference, and global dose normalization.

The dose delivery across the junction was verified by irradiating both plans using the Delta4 detector in the same measurement session. For both plans, we positioned the Delta4 at the lateral and sagittal green laser position and longitudinally in the plan junction markers, due to the red to green laser separation limit of 15 cm for. The distance from the longitudinal green laser position to the Delta4 was measured in the DQA module and applied at setup. After irradiation of the upper plan, the detector was rotated and aligned to the lasers for the lower plan and subsequently irradiated in the same measurement session. The planned dose for the upper and lower body was manually summed using Python.

## 3 RESULTS

### 3.A Phantom

**Figure 3:**
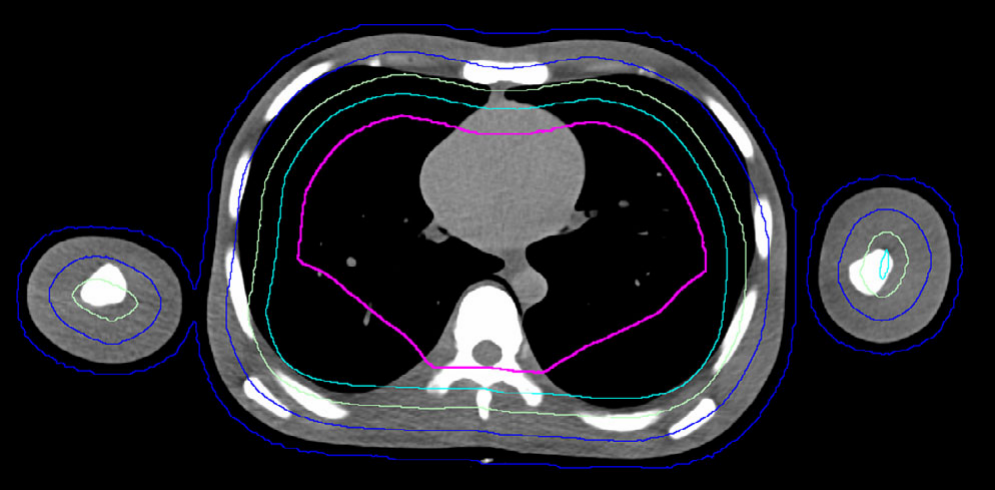
Transverse slice of the PBU‐60 phantom with planning target volume (blue) and several blocking structures (green, turquoise, pink) adjusted for increased target coverage in the back region, close to the vertebral column.

Doses to OARs are presented in Table 1. The optimization time for 500 iterations ranged between 4 and 6 h with a GPU‐assisted dose calculation engine. The beam on‐times for the final plan were 31 and 19 min, for the upper and lower body, respectively. In the optimization, some adjustment of the blocking structure was required to compensate for the flat back of the phantom (see Fig. 3). This adjustment resulted in higher dose to the lungs of the phantom, due to the thin thorax wall of the PBU‐60 phantom (6 mm). For the patients, this was corrected for by immobilizing the back in a rounded position.

**Table 1:** Dose to the PBU‐60 phantom (Gy) with a prescribed dose of 12 Gy in 6 fractions, patient 1 with a prescribed dose of 12 Gy in 6 fractions, and patient 2, with a prescribed dose of 20 Gy in 10 fractions

Structure	Phantom			Patient 1			Patient 2		
	D_2%_	D_98%_	D_mean_	D_2%_	D_98%_	D_mean_	D_2%_	D_98%_	D_mean_
Bladder	1.9	1.1	1.4	1.1	0.8	0.9	13.4	2.1	7.5
Body	12.4	0.4	6.4	12.7	0.8	6.0	1.5	21.0	10.9
Bone	12.2	0.6	6.1	12.1	0.8	4.2	20.2	1.4	7.7
Bowelbag	7.9	0.7	1.6	5.3	0.7	1.3	1.4	3.5	1.9
Brain	11.1	0.6	3.1	9.7	0.7	2.6	15.0	1.4	4.5
CTV 5 mm	12.9	10.5	11.9	13.0	10.9	12.1	22.0	16.5	20.0
Eye (left)	9.9	0.8	4.4	7.7	1.4	4.6	20.3	7.9	17.4
Eye (right)	10.0	0.8	3.9	7.6	1.4	4.6	20.6	7.9	17.6
Heart	1.5	0.7	0.9	1.8	0.9	1.1	2.6	1.7	2.0
Kidney (left)	1.5	0.7	1.0	1.8	0.9	1.3	2.6	1.5	1.9
Kidney (right)	1.7	0.7	1.0	1.6	0.9	1.2	2.3	1.4	1.8
Lens (left)	7.2	5.1	6.7	4.8	3.6	4.0	19.8	20.2	20.0
Lens (right)	7.1	4.6	5.7	4.8	3.8	4.1	20.0	20.5	20.3
Liver	3.5	0.6	1.1	7.3	0.9	1.6	2.3	1.5	2.0
Lung (left)	9.6	0.9	3.3	5.7	1.1	1.9	10.9	2.1	3.3
Lung (right)	10.9	0.9	4.1	6.8	1.0	1.9	10.9	2.0	3.1
Oral cavity	9.3	0.9	4.6	1.1	0.8	3.4	18.5	1.8	5.5
PTV	13.0	9.6	11.9	12.9	10.1	11.9	22.0	16.4	20.0

CTV: clinical target volume; PTV: planning target volume.

**Figure 4:**
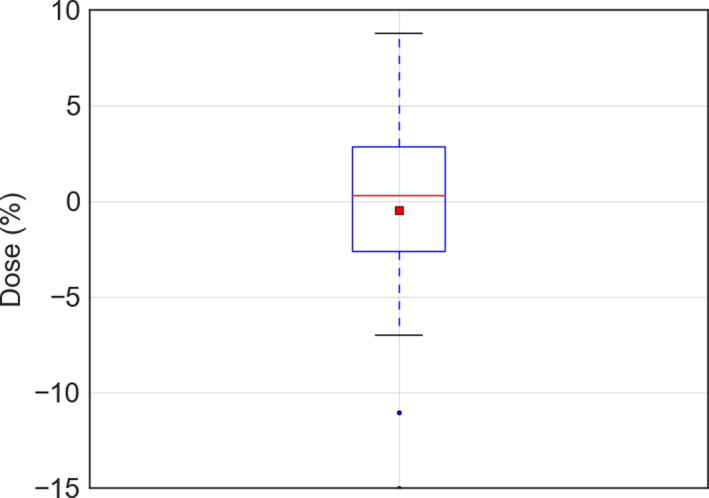
The dose measured with electron beam therapy 3 film on the PBU phantom relative to that predicted by the treatment planning system recalculated without the virtual bolus, shown as a box‐and‐whisker plot, where the red line shows the mean of 23 measurements, median (red line), 1 SD (box) and 95% confidence interval (outer line) as well as outliers (black point).

**Figure 5:**
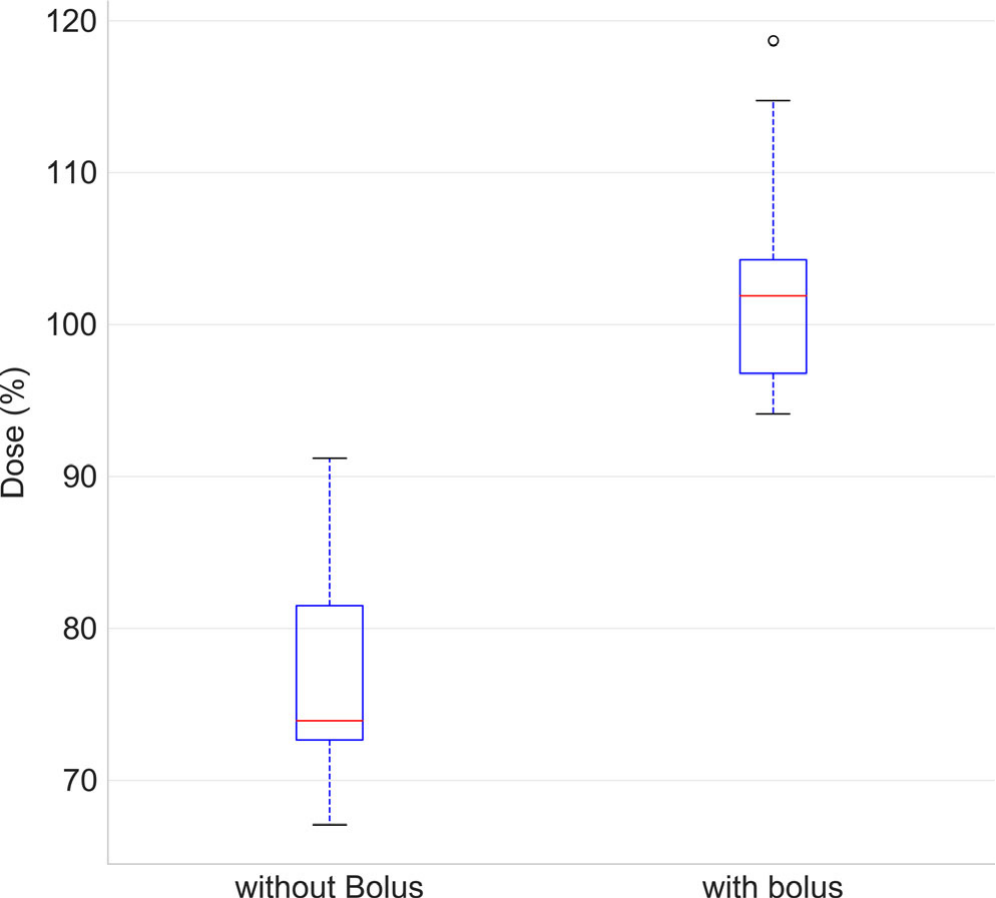
Measurements with and without wet suit using electron beam therapy 3 film on the PBU‐60 phantom presented as a box‐and‐whisker plot. The median (red line), as well as first and third quartile (box) and 1.5 times past the interquartile range (outer line) is plotted with outliers (black points). Dose is presented as percentage of prescribed dose.

Verification of the dose to the surface of the whole body phantom using EBT3 film agreed well with the dose to the PBU‐60 phantom calculated without the virtual bolus. The results indicate that the dose calculated in the TPS provides a good approximation of the delivered skin dose. When using the virtual bolus and neoprene for build‐up, the average dose difference between TPS and film measurements was −0.6% (SD = 3%; Fig. 4). The paired measurements, with and without wet suit, on the PBU‐60 phantom showed a significantly higher surface dose with the 7 mm neoprene bolus than irradiation without bolus (Wilcoxon signed‐rank test, *P* < 0.05; Figure 5). Measurements at 0 cm depth with and without a 7 mm sheet of neoprene placed on a Solid Water slab resulted in a 57% higher dose with the bolus, confirming the advantage of using neoprene as bolus material. Central dose measurements using two A1SL ion chambers in the cylindrical Tomotherapy phantom showed good agreement with the values given by the TPS; being within 2% of the TPS values at all 6 measured points. This indicated that the measurements are correctly represented by the superimposed convolution algorithm in the TPS, even though the measured points are far away from the interaction site (2–15 cm) of the primary target.^15^ Measurements with the Delta4 diode array detector were performed at three different positions to cover different areas of the treatment plan, including the junction position of the plans, yielding gamma pass rates of 90%, 93%, and 97%, with global dose normalization. Consequently, the delivered dose was generally in good agreement with the planned dose at all measured positions, and within the pass rate criteria used at our clinic (90%). The dose across the junction region was evaluated with a structure created as a copy of the PTV, extending 2 cm cranially and 2 cm caudally of the junction markers. Dose to the structure was D_98%_ of 11.2 Gy, D_mean_ of 12.8 Gy, and D_2%_ of 14.2 Gy.

The setup robustness test, performed by shifting the PBU‐60 phantom in six directions yielded an average D_98%_ of 10.4 Gy (SD = 0.3 Gy) and D_2%_ of 13.4 Gy (SD = 0.1 Gy), indicating that this setup, combining a virtual bolus of 8 mm with a density of 0.4 g/cm^3^and a physical neoprene bolus provides robust treatment for positioning errors of up to 10 mm. Recalculating the plan with different densities of the virtual bolus resulted in differences in the average dose to the CTV of −0.2 %, 0.2%, and 2.7%, for densities of 0 g/cm^3^, 0.4 g/cm^3^, and 1 g/cm^3^, respectively, justifying the use of a virtual bolus with a density lower than that of water as proposed by Moliner et al.^13^

**Figure 6:**
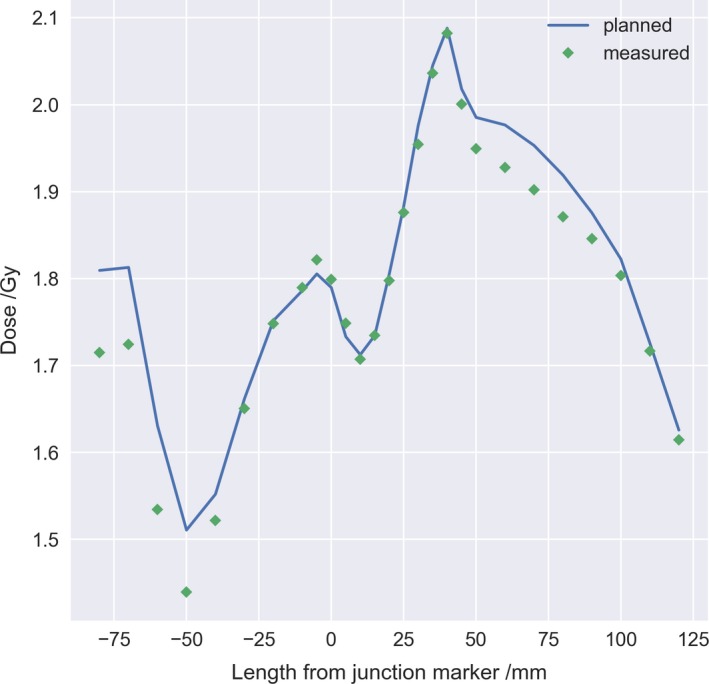
Longitudinal line dose profile across the plan junction (0 mm) for both plans recalculated on the Delta4 phantom (blue line) and summed manually as compared to the measured dose (green diamond) with the Delta4 rotated for measurement of the lower body plan.

**Figure 7:**
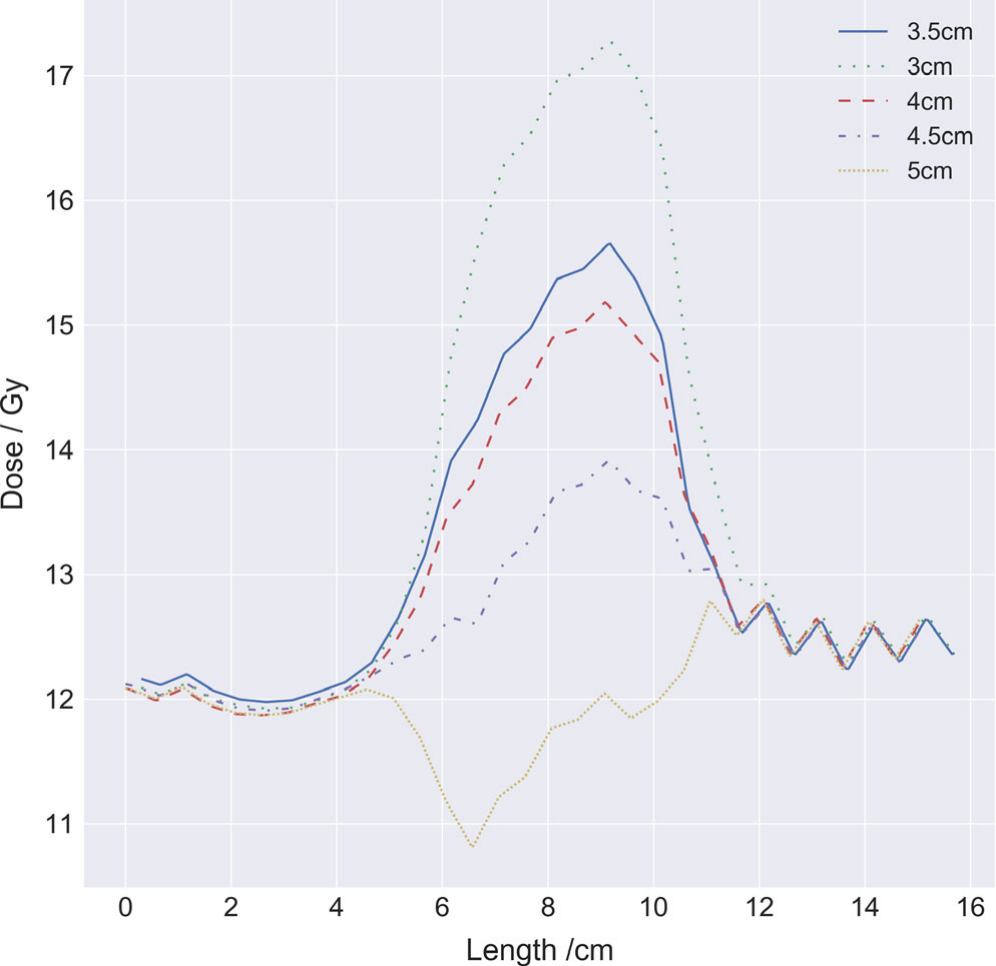
Longitudinal line dose profiles for different total length of junction structures, between 3 and 5 cm in increments of 0.5 cm. The upper and lower body plan was summed in Eclipse and the line profile was acquired at 3 mm depth centered lateral over the junction marker.

The extension of the junction structure between the lower and upper body of the PBU‐60 was adjusted until an acceptable dose distribution was achieved, which was 4.5 cm for the phantom. The resulting test of the robustness, with repositioning and recalculation of the upper body and subsequent summation in Eclipse with the lower body, yielded a D_95%_ range of 11.5 to 11.7 Gy, and D_5%_ range of 14.2 to 14.7 Gy for 5 mm translational offset. For 10 mm translations, the D_95%_ dose ranged from 8.9 to 12.1 Gy and D_5%_ ranged from 12.2 to 14.8 Gy. The lowest doses found were in all cases from longitudinal setup errors. Line profiles from measurements with Delta4 as compared to planned dose recalculated on the Delta4 and summed over the junction region are reported in Fig. 6, and line profiles of the junction for different total length of the junction structure in Fig. 7.

### 3.B Patient

Based on the experience from the immobilization of the PBU‐60 phantom, the patients were immobilized with the back in a laterally curved position to better facilitate tangential irradiation in the optimization.

**Figure 8:**
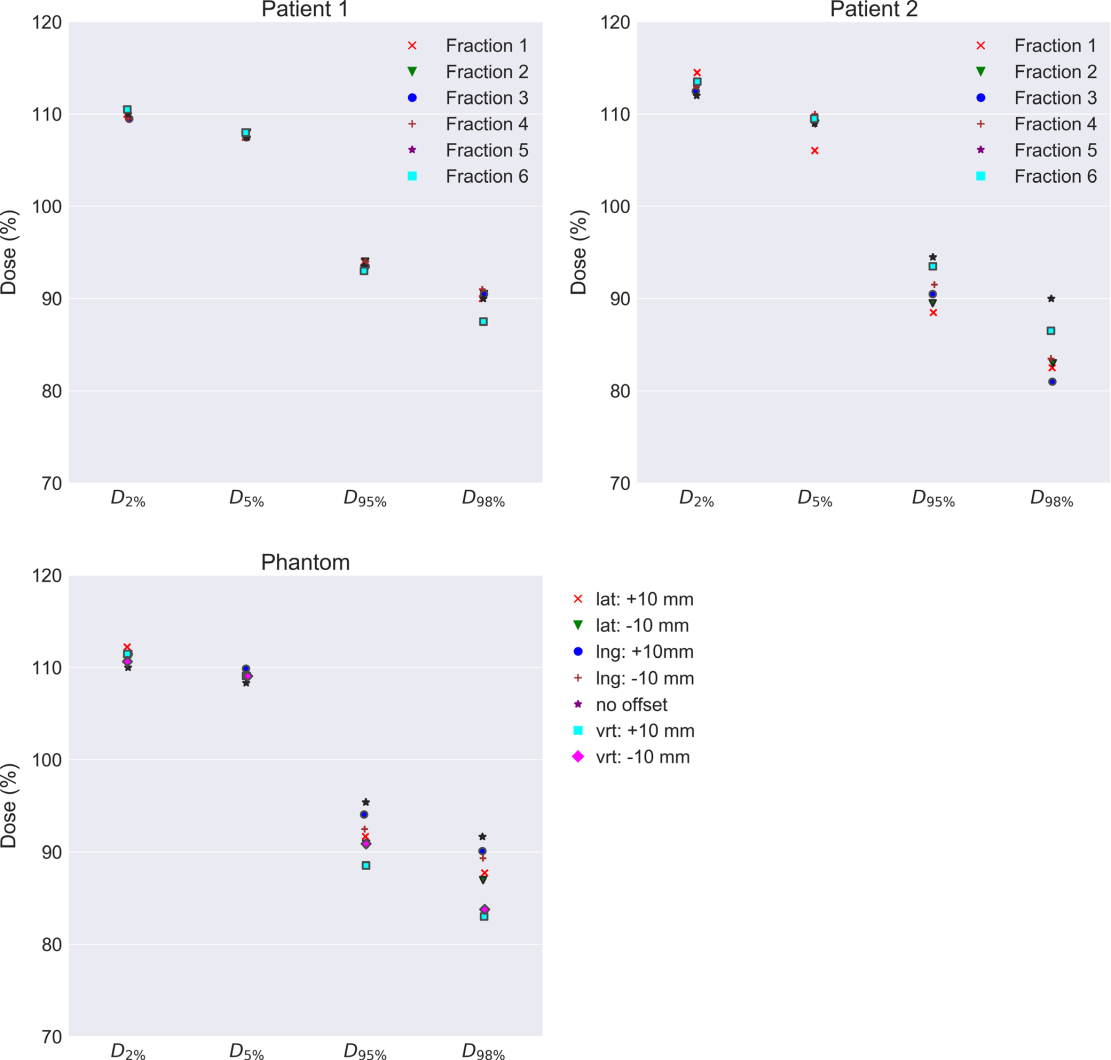
Fraction dose delivered recalculated on megavoltage computed tomographic daily imaging for the first 6 fraction for patient 1 and 2, compared to robustness test data of the setup position using the PBU‐60 phantom. For the phantom, the clinical target volume (CTV) coverage for the upper body is plotted for 10 mm offset in each direction against coverage with no offset. For the patients, CTV coverage for the upper body is plotted for the first 6 fractions.

**Figure 9:**
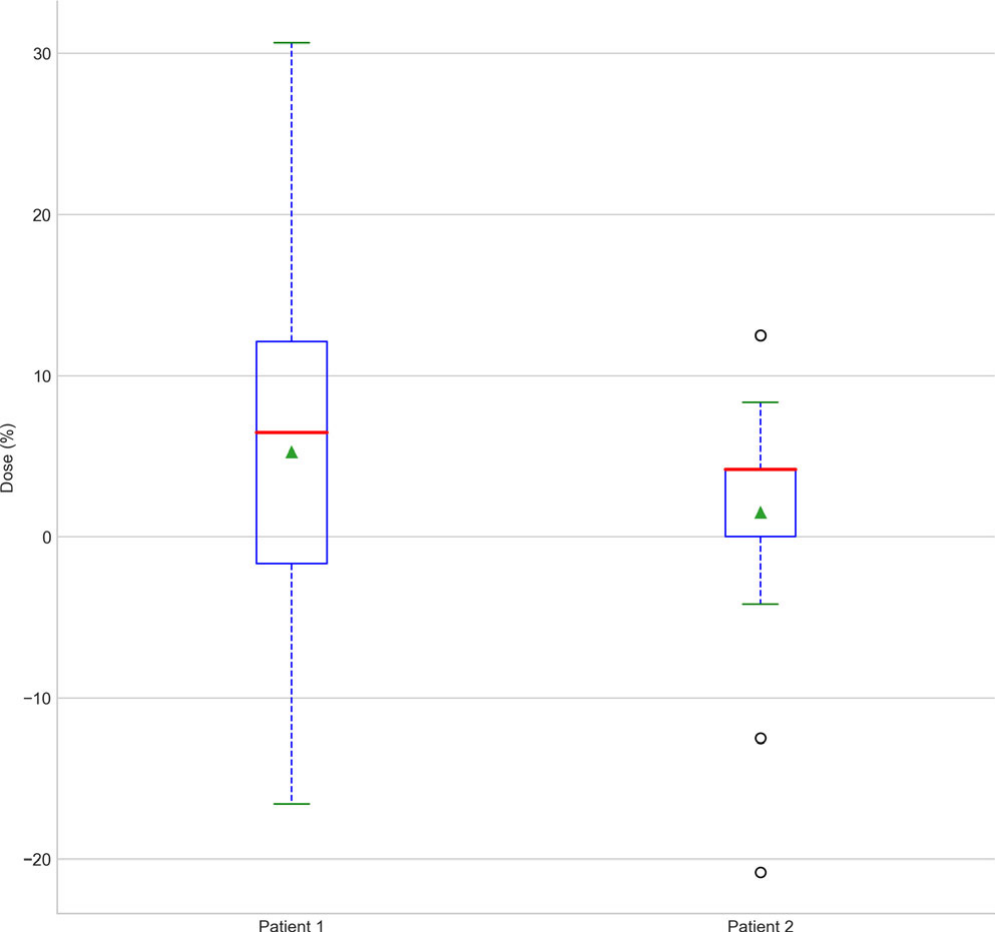
Measured dose with electron beam therapy 3 film for patient 1 and 2 at the first fraction. Dose is plotted as the difference to prescribed fraction dose. Data are shown as a box‐and‐whisker plot, where the red line shows the mean of 23 measurements, median (red line), mean (green triangle), 1 SD (box), and 95% confidence interval (outer line) as well as outliers (black point).

For both patients, the six‐first fractions were recalculated based on daily MVCT images and compared to the robustness calculations performed with the PBU‐60 phantom (Fig. 8). In addition, *in vivo* film dosimetry corresponded well with dose calculated in TPS, with a mean difference from TPS of 5.3% (SD = 11.9%) and 1.5% (SD = 9.0%) for patient 1 and 2 respectively (Fig. 9). Robustness test of the junction dose performed on the first patient yielded an average D_98%_ of 10.8 Gy (SD = 0.2 Gy) and D_2%_ of 13.4 Gy (SD = 0.2 Gy) for 5 mm translations.

**Figure 10:**
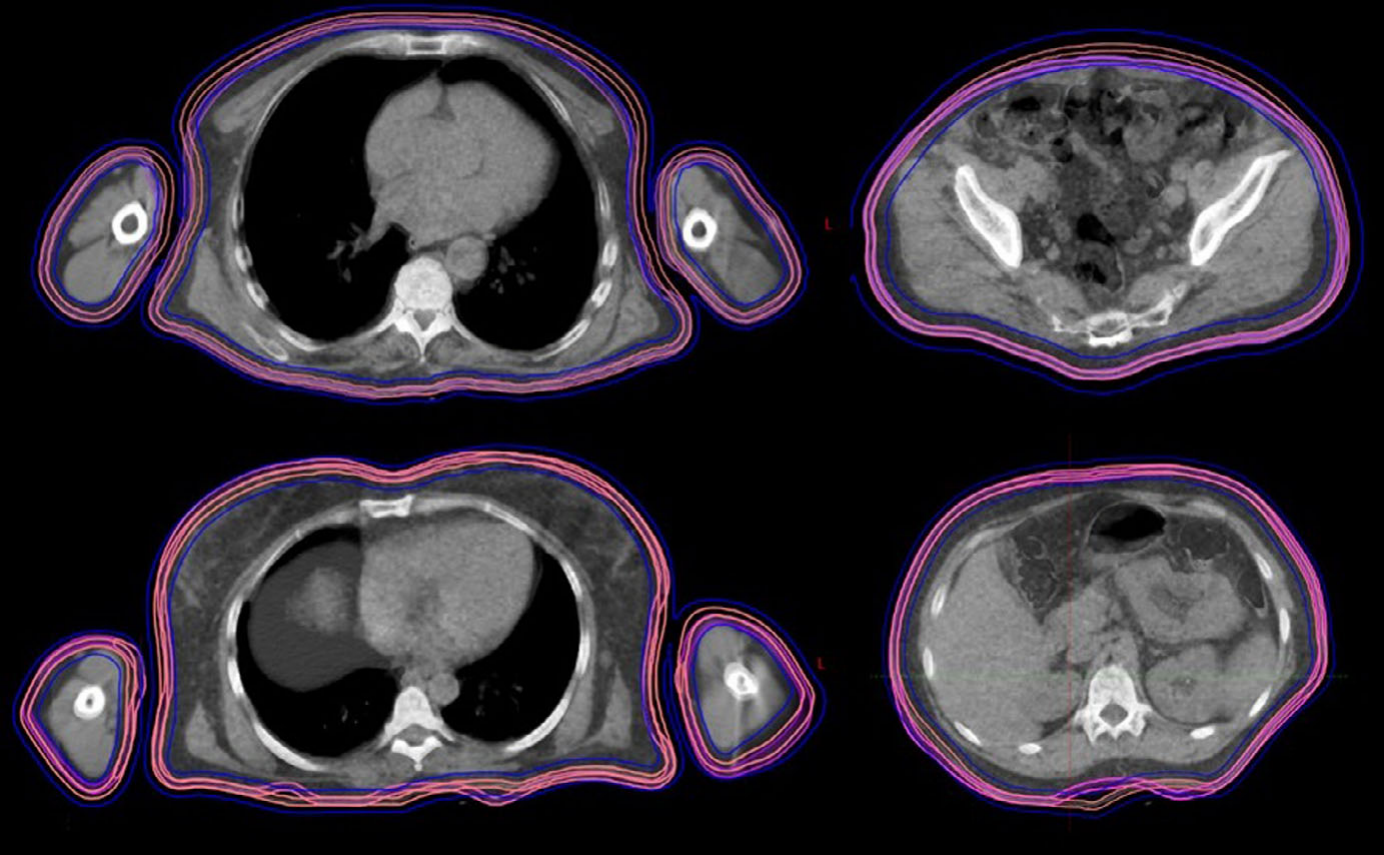
Daily megavoltage computed tomographic (MVCT) from the first fraction compared to planning computed tomography (CT) for patient 1 and 2. The CT and daily MVCT are overlayed with 50% transparency. Transversal slices for abdomen and thorax are displayed with original clinical target volume (CTV) (pink) with CTV for daily MVCT (magenta) and planning target volume (blue) from planning CT.

Both patients could put the wetsuit on within a few minutes, with no notable effort. The fit for patient 2 was not optimal which was compensated for by taping air gaps to achieve a snug fit. Figure 10 shows examples of planning CT and MVCT image registration at the time of treatment. The CTV coverage was regarded adequate despite daily variations in fit of wet suit and skin folds. The total beam on time for the patients were 92 and 54 min for patient 1 and 2 respectively, with the length of the patient the deciding factor, 190 vs 165 cm. Both patients completed their treatment as planned and tolerated the treatment well with the second patient given only a mild sedative.

## 4 DISCUSSION

### 4.A Bolus

The surface dose measured with EBT3 film agreed well with that provided by the TPS for both the PBU‐60 and the TomoTherapy phantom. The difference in the surface dose found for paired measurements with and without the neoprene bolus was significant, justifying the use of a virtual bolus as well as a physical bolus for total skin delivery, despite the fact that neoprene is not a standard material, and the lack of water equivalence in the material. The results quantify the difference with using neoprene as bolus and stands in contrast to other studies that did not find it necessary to use bolus for skin irradiation.^8^, ^16^ The dosimetric advantage to a non‐bolus treatment is clear, any attempt at optimizing or deliver photons to the skin performs better with bolus added.

### 4.B Robustness

The robustness test showed that calculated and delivered dose corresponded well for displacements up to 10 mm, despite a CTV‐to‐PTV margin of only 5 mm. Underdosage to the skin of up to 20%, and an increase in average dose to the target, have been reported previously,^8^ depending on the parameters of the virtual bolus. When a thick high‐density virtual bolus is used, the lack of corresponding physical bolus at treatment delivery causes overdosage to the skin and to a depth up to a few centimeters. When no bolus is used, physical or virtual, underdosage of the skin is likely due to build‐up, and large dose deviations can be expected with normal patient displacements since optimization to the lower density (air) causes a high fluence. The measured and calculated skin dose agreed within a few percent in this study, even with displacements of up to 10 mm, by careful selection of the virtual bolus density and thickness (0.4 g/cm^3^, 8 mm) and planning parameters. The largest setup uncertainties in the phantom measurements were found around the arms, due to the difficulty of correct alignment caused by the limited field of view of the TomoTherapy MVCT (40 cm), that is, parts of the patient more than 40 cm from the isoaxis as seen in a transversal plane are not included in the MVCT. This issue may be resolved by using, for example, an external surface scanning system. Predicted doses to OARs in this study are similar to, or slightly higher than, those reported previously.^7^, ^8^ These differences can be attributed to differences in CTV‐to‐PTV margins or differences in phantom anatomy.

The methodology for matching the junction dose of the treatment plans worked well for the treated patients and the phantom, but the dose distribution was more heterogeneous than in other parts of the target. The effect of using an overlapping structure works similar to cropping the PTV, but the benefit of a junction structure was the ability of adjusting the overlap region and still get the dose coverage correct in the PTV used for optimization. Improvement such as creating an extended optimization region with several optimization structures to better control the dose fall‐off, would probably improve the dose homogeneity and robustness across the field junction. Line dose profiles acquired longitudinally for different junction lengths showed that a difference in junction length may result in an unproportional response in overlap dose, probably attributed to a combination of the amount of blocking and the gantry angle position at the current slice. Line dose profiles for measured dose compared to planned dose agreed well, showing the highest deviation at maximum and minimum dose owing to the difference in resolution, were the detectors in the Delta4 have 5 mm dispersion and the plan was calculated with a grid of 2.3 mm.

The first two patients were very different regarding BMI and fit of wet suit. Experience showed that a snug fit is vital to keep the workflow easy and daily variations to a minimum. For patient #2, loose skin and fatty tissue caused different skin folding from day to day. However, no repositioning was needed, indicating that the skin folds was considered within tolerance. These issues affected the delivered dose as seen by the daily fraction calculations in and should be addressed by focusing on proper fitting of the wet suit. Another learning experience was to keep the arms close to the body. Although arms positioned farther from the thorax facilitate optimization of the arm circumference, it counteracts the optimization of dose homogeneity to the arms. As shown by *in vivo* film measurements, the measured dose to the skin was closer to the predicted (1.5%–5.3%) than reported in other studies with different physical and virtual bolus.^7^, ^8^ Previous studies on skin doses in the tomotherapy TPS has shown an overestimation of the calculated dose by approximately 9%.^17^, ^18^ The number of patients in the study makes this a first experience, and more patients need to be added to draw any general conclusions. A clinical study is in the planning phase.

### 4.C Comparison to standard treatment

TSEBT is today regarded as the standard treatment of mucosis fungoides and in comparison, TSI using HT is a lengthy and complex treatment. The average beam on time was 73 min for our two patients and the in‐room time between 3 and 3.5 h. In comparison, TSEBT is given twice daily, and with separate x‐ray treatments given to the hands, the soles of the feet and scalp. Piotrowski et al.^19^ argued that the rotational TSEBT requires less time, but did not include the time for added extra x‐ray fields necessary to cover scalp and other areas not covered in the electron irradiation in their estimation. Our experience from other complex treatments is that a new clinical routine takes a number of patients to set, and treatment time can very likely be reduced with surface guided positioning. For comparison, at our clinic, total marrow irradiation with HT took almost 3 h for the first patient and the fastest fraction treated after 23 patients is closer to 1 h, partly as a result of surface guided positioning and an efficient clinical routine.

Doses to organs at risk are generally below clinically used dose constraints, but a comparison with TSEBT is not possible as, to the best of the authors' knowledge, doses to organs at risk have not been published for TSEBT. Slightly higher doses are expected to deep‐lying organs in TSI with HT compared to TSEBT, which is a trade‐off. In contrast, high robustness and a homogeneous target coverage can be achieved on a single treatment occasion using Tomotherapy.

Similar to results reported by Buglione et al.^16^ we believe TSI with TT to be a complement to electron treatment and in certain cases where treatment with Tomotherapy could be beneficial. In addition, since TSI with HT is an image guided technique, problems that may arise during treatment can be evaluated by dose recalculation or re‐optimization of the treatment plan. Previously treated areas and organs at risks can be avoided, and simultaneous integrated boost to for example, plaque areas can be implemented. The results from this study can be of use when treating patients with partial irradiation of large areas, especially of convex shape such as the scalp^20^ or melanoma.^21^ Furthermore, this technique may be an alternative to centers where electron therapy is not available.

### 4.D Film dosimetry

The EBT3 film is an established and appropriate dosimetry system for surface dose measurement.^22^, ^23^, ^24^ It has a low angle dependence and stable response over a wide dose and energy range, especially when used with the FilmQApro scanning system, where all color channels can be evaluated. The largest uncertainty stems from positioning accuracy, that is, the problem to correctly assess the points of measurement of the films in the TPS for correct dose comparison.

### 4.E Conclusions

The presented technique was shown to be feasible and robust to deliver for both phantoms and for two individual patients. We believe that TSI with tomotherapy may an alternative for centers without electron beam capability, if a more homogenous dose is desirable, or for partial skin irradiation were electron therapy for any reason is not feasible.

## CONFLICT OF INTEREST

Department of corresponding author have an ongoing research agreement with Accuray Inc. which includes funding.
